# Supplementary material for: A new metoposaurid (Temnospondyli) bonebed from the lower Popo Agie Formation (Carnian, Triassic) and an assessment of skeletal sorting
Source: PLoS One. 2025 Apr 2;20(4):e0317325. doi: 10.1371/journal.pone.0317325 (PMC11964259; doi:10.1371/journal.pone.0317325)
Supplement: S1 File — (DOCX) [file pone.0317325.s004.docx]

**Supplemental Text and Figures for**

A new metoposaurid (Temnospondyli) bonebed from the lower Popo Agie Formation (Carnian, Triassic) and an assessment of skeletal sorting

Aaron M. Kufner*^1,2^; Max E. Deckman^3^; Hannah R. Miller^2,4^; Calvin So^5,6^; Brandon R. Price^2^; David M. Lovelace*^1,2^

^1^Department of Geoscience, University of Wisconsin-Madison, Madison, WI, 53706;

^2^University of Wisconsin Geology Museum, Madison, WI, 53706;

^3^Department of Geology, University of Georgia, Athens, GA, 30602;

^4^College of Design, University of Kentucky, Lexington, KY, 40508;

^5^Department of Biological Sciences, George Washington University, Washington, DC, 20052;

^6^Negaunee Integrative Research Center, Field Museum of Natural History, 1400 S Lake Shore Dr, Chicago, IL, USA

*Corresponding authors: Aaron M. Kufner; David M. Lovelace

Email: akufner@wisc.edu (AMK); dlovelace@wisc.edu (DML)

## **Extended Methods for Skeletal Sorting**

Skeletal elements were identified from Nobby Knob (NK; S1 Table), Elkins Place (EP; S2 Table), and Site XIII (S3 Table). Many of the NK specimens are uncatalogued, but the field and lab numbers used in S1 Table and S1–S6 Figs will be associated with final catalog numbers (i.e. UWGM). Both NK and EP have several disarticulated cranial bones that if each was counted as a single Voorhies Group III element, it would skew the data significantly. It is unclear if the remains were disarticulated pre- or post-deposition, so all cranial remains including elements of articulated skulls were separated into their constituent parts (e.g., left and right premaxilla, left and right maxilla, etc.). The frequency of each element was counted using S2 File.R to determine both the total number of each representative element and also a minimum number of individuals (MNI). The summed list of elements was then exported, and the proportion of cranial/mandibular remains was calculated manually by dividing the count of all cranial/mandibular bones by the expected number based on MNI.

Our assignment of skeletal elements to Voorhies groups (Table 2) follows previous studies (Lucas et al., 2010, 2016; Rinehart et al., 2024), however, none of these places an estimate on the total count of each element in a metoposaurid skeleton. Rinehart and Lucas (2016) used a similar framework with a bonebed of the capitosaurid *Eocyclotosaurus appetolatus* providing estimates of skeletal elements. These estimates were used as our basis with some modifications to the counts for the “forearms/legs” from 12 to eight because humeri and fibulae were counted separately (table 1: Rinehart & Lucas, 2016). Neural arch and hemal arch counts were also split and counts were adjusted based on previous descriptions of more completely known metoposaurids (Dutuit, 1976; Konietzko-Meier et al., 2020; Sulej, 2007).

## **Scanning Electron Microscope (SEM) Methods**

An uncatalogued external mold of a bivalve from the field jacket NK19-C2-709.3 was selected for additional analysis due to the presence of a slightly darker stain on the mold. Energy dispersive spectroscopy (EDS) was performed on the sample in the Hitachi S-3400 N Variable Pressure SEM (S8 Fig). A 15 kV electron beam was used in variable pressure mode to elicit characteristic X-rays that were captured using 15 seconds of live time per spectrum by the Oxford Instruments x-act EDS detector. The collected spectra were analyzed by Oxford’s AZtec 4.1 software yielding normalized weight percent. Light elements such as carbon are at the low end of the detectable range of X-rays with these instruments.

## **Systematic paleontology**

BIVALVIA Linnaeus 1758

UNIONOIDA Gray 1854 (*sensu* Newell, 1965)

*ANTEDIPLODON* Marshall 1929 (*sensu* Good, 1998)

*ANTEDIPLODON* sp.

(Fig 6)

**Referred specimens.**—UWGM 7567, 7571–7573, 7584.

**Description and rationale for taxonomic assignment.**—Unionoid bivalves have been reported from the Popo Agie Formation and were considered similar to *Unio dumblei* Simpson 1895 (=*Antediplodon dumblei*, *sensu* 80) from the Dockum Group of Texas (Berry, 1924). None of the specimens from the NK locality reveals the entire morphology of the external valve surface, but some taxonomically informative anatomy can be determined in aggregate. The annuli are apparent, and the nearly complete external molds have an oblong ovate shape with an abrupt anterior end (Fig 6). Several riblets radiate from the umbo, and there are at least 17 ridges in a “pseudo-radial” pattern (*sensu* Zieritz et al., 2015) although none of the specimens reveals the umbo in its entirety. Similar to *Antediplodon dockumensis* Simpson 1895 (*sensu* Good, 1998), all specimens lack lirae between the annuli. Species of *Antediplodon* can also be differentiated by the thickness of the shell which cannot be assessed here due to the nature of preservation.

**Remarks.**—All bivalves from the NK locality are preserved as molds, all of which appear to be external molds due to the presence of only external morphology on both the positive and the negative relief (see Fig 6A–B). Bivalve molds are found below, within, and above the bonebed, but they appear more common in the layers underlying the bonebed. There may be additional bivalve taxa at NK, but only more complete specimens will be conclusive. UWGM 7567 is notable in lacking any apparent riblets radiating from the umbo similar to *Triaslacus* Bogan & Weaver 2012. UWGM 7584 is anteroposteriorly elongate with at least 7 riblets radiating from the umbo (Figs 6C–D). In addition to the specimens included here, there are dozens of uncatalogued bivalve impressions from the NK locality all exhibiting similar morphology to those described.

OSTEICHTHYES Huxley 1880 (*sensu* Nelson et al., 2016)

ACTINOPTERYGII Woodward 1891 (*sensu* Goodrich, 1930)

REDFIELDIIFORMES Berg 1940 (*sensu* Schaeffer, 1984)

REDFIELDIIDAE Berg 1940 (*sensu* Hutchinson, 1973)

REDFIELDIIDAE gen. et sp. indet.

(Fig 7)

**Referred specimens.**—UWGM 7575, 7576, 7579, and 7586.

**Description and rationale for taxonomic assignment.**—Actinopterygian remains from this site may be referable to Redfieldiidae although only the mold of an element that most closely resembles a redfieldiid supracleithrum (e.g. figs 5, 8b–f: Gibson, 2018) is preserved with most of the element lost during preparation (Fig 7F–G). Several isolated ganoid scales, conical teeth, and fragments of similar bone were recovered. The ganoid scales are diamond-shaped and lack any apparent ornamentation (Figs 7C–D) similar to *Lasalichthys otischalkensis* Gibson 2018. A fragmentary dentigerous element appears to show teeth angled in multiple directions (Fig 7E) much like the characteristic morphology of the rostral bone of Redfieldiiformes (Gibson, 2018).

**Remarks.**—Isolated actinopterygian remains have been recovered from the bonebed and appear more common in the underlying sediment, but they are rare and fragmented compared to the metoposaurid remains. It is unclear if a probable actinopterygian fin from NK (Figs 7A–B) is from a redfieldiid or an as yet unidentified actinopterygian.

TETRAPODA Jaekel 1909

TEMNOSPONDYLI von Zittel 1887–1890 (*sensu* Schoch, 2013)

METOPOSAURIDAE Watson 1919 (*sensu* Buffa et al., 2019)

*BUETTNERERPETON* Gee & Kufner 2022

*BUETTNERERPETON BAKERI* Case 1931 (*sensu* Gee & Kufner, 2022)

(Fig 5)

**Referred specimens.**—See S1 Table for complete list.

**Description and rationale for taxonomic assignment.**—The vast majority of vertebrate remains from the NK locality are referable to Metoposauridae, and the more complete cranial remains are indistinguishable from *Buettnererpeton* *bakeri* Gee & Kufner 2022. Some of the salient features of the skull that demonstrate this taxonomic assignment are: (1) lacrimal separated from the orbital margin by contact of the prefrontal and the jugal, (2) deep otic notch, (3) well-developed tabular horn, and (4) anterior margin of orbit posterior to anterior margin of interpterygoid vacuity. Additional features of the postcranial skeleton (not figured) that demonstrate this taxonomic assignment are: (1) large areas of reticulate ornamentation on both the interclavicle and the clavicle and (2) a sensory groove along the ventral side of the posteromedial margin of the clavicle.

**Remarks.**—Specimens from the type locality of *Buettnererpeton bakeri* (Elkins Place) are primarily from similar-sized individuals (midline skull length ~30 cm) with the exception of at least one fragmentary, large individual (Gee & Kufner, 2022). The NK locality preserves a relatively wide size range of nearly complete skulls (<20 – ~40 cm midline length) as well as a greater representative sample of the postcranial skeleton than the type locality of *B. bakeri*.

DIAPSIDA Osborn 1903

ARCHOSAUROMORPHA Huene 1946 (*sensu* Benton, 1985)

ARCHOSAUROMORPHA gen. et sp. indet.

(Figs 8A–B)

**Referred specimens.**—UWGM 7569, 7570, and 7585.

**Description and rationale for taxonomic assignment.**—Three isolated, shed archosauromorph teeth were recovered from the NK bonebed. The two most complete teeth are shown in Figs 8A–B. One tooth (Fig 8A; UWGM 7585) is recurved and conical with fine serrations (=denticles) on its mesial and distal margins. Another tooth (Fig 8B; UWGM 7569; possibly a maxillary or posterior dentary tooth) is subtriangular in labial and lingual views and mediolaterally compressed. Both the mesial and distal margins are slightly convex and lined with serrations, and the tooth is not expanded at its base. The presence of both mesial and distal serrations suggests archosauriform affinities (Nesbitt, 2011), however, some malerisaurine azendohsaurids convergently acquired tooth serrations (Marsh et al., 2022; Nesbitt et al., 2022) restricting our assignment of isolated serrated teeth to Archosauromorpha. Previously described archosauromorphs from the Popo Agie Formation include phytosaurs, a poposaurid, a loricatan, and two dinosauriforms (Table 1).

ARCHOSAURIFORMES Gauthier et al. 1988

PHYTOSAURIA Meyer 1861 (*sensu* Doyle & Sues, 1995)

PARASUCHIDAE Lydekker 1885 (*sensu* Kammerer et al., 2016)

PARASUCHIDAE gen. et sp. indet. (Figs 8D–J)

**Referred specimens.**—UWGM 1995, 7578.

**Description and rationale for taxonomic assignment.**—Fragments of a phytosaur mandible (UWGM 1995, 7578) were recovered from above the NK bonebed or as weathered fragments around the hill. UWGM 1995 is made up of several fragments collected as “float” including a segment of a right dentary. The fragment of dentary bears at least seven alveoli and a prominent median platform that rises above the level of the lateral margin of the element. This segment is D-shaped in cross section similar to the cross section of the anterior portion of UWGM 7578. (Figs 8I–J). The medial surface of the dentary has posteriorly dipping interdigitations of the symphyseal plate (Fig 8E). The largest fragment is made up of the angular and the splenial from the portion of the mandible ventral to the mandibular fenestrae (Figs 8I–J). The labial surface of this element is ornamented with raised ridges at its posterior extent. The lingual surface is smooth, flat, and lacks any symphyseal suture morphology such as that seen in UWGM 1995. There is a depression on the lingual surface of the angular of UWGM 7578 at the posterior extent of the preserved element (Fig 8J) bounded ventrally by a medially inflected ridge.

**Remarks.**—These fragments were all recovered *ex situ* and are most likely from a horizon above the primary NK bone layer, exhumed during removal of overburden. The bone-bearing horizon above the primary bone layer of NK could not be located, however, the horizon can be stratigraphically constrained to within 5 m above the NK bonebed, below the purple-ocher transition zone.

## **Supplemental Figures and Figure Captions**


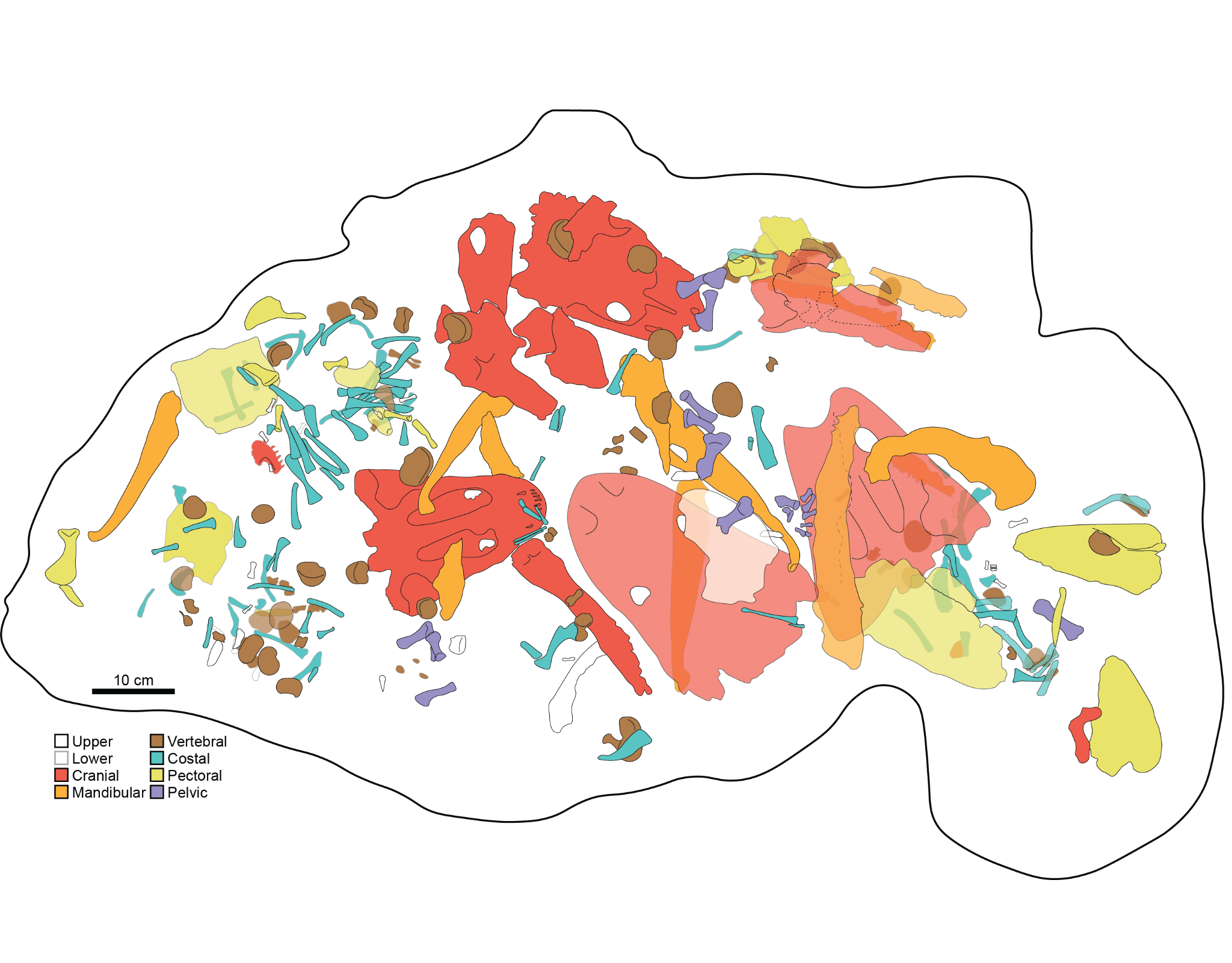

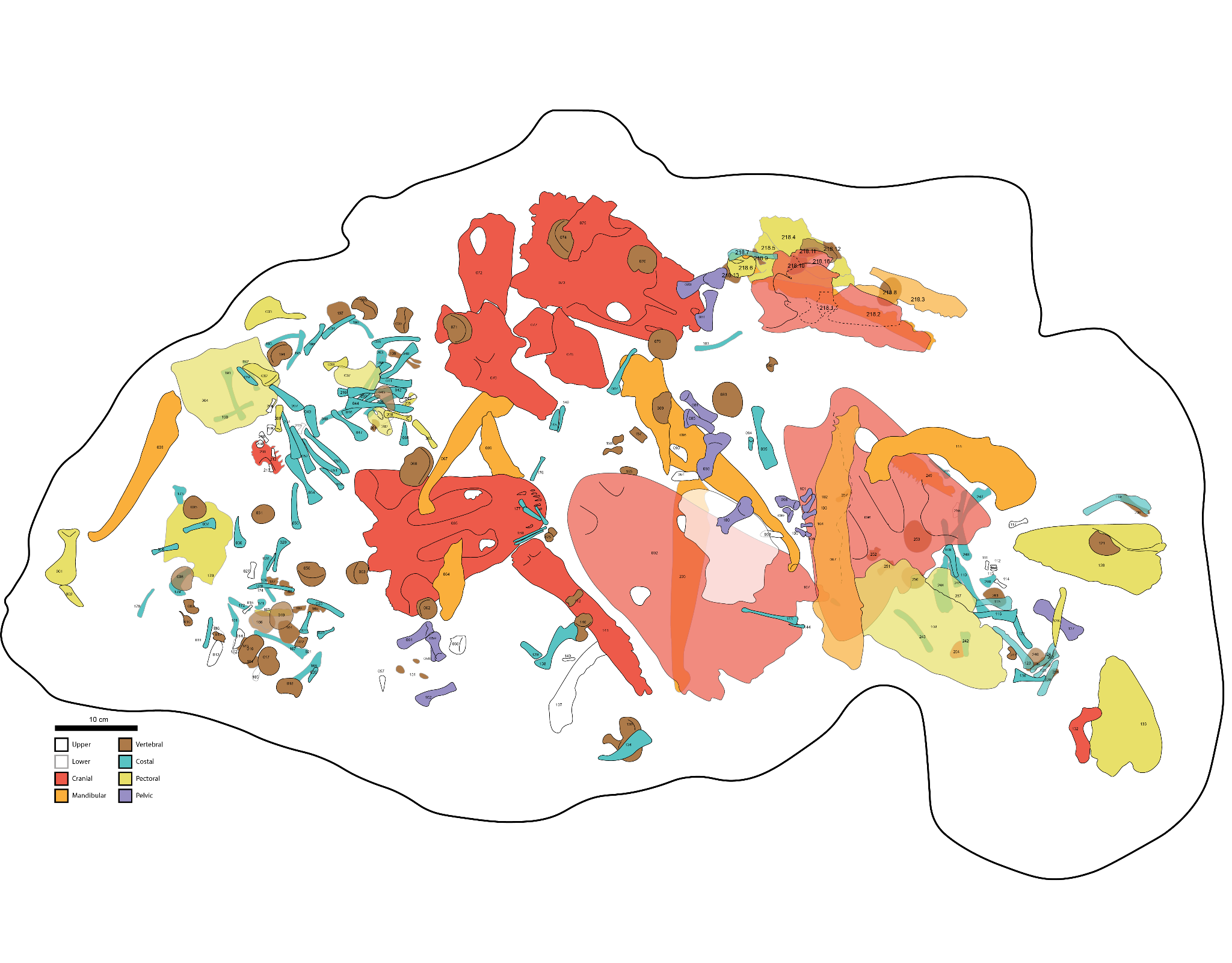


**S1 Fig. Schematic drawing of field jacket NK16 J10 3-5.** High resolution jacket map as included in quarry map (top) and with numbers that correspond to “Prep Lab Number” in S1 Table (bottom). Some elements are slightly transparent to show underlying bones used in the azimuthal and/or total element count analyses. Colors indicate anatomical position as follows: red=cranial, orange=mandibular, brown=vertebral, blue=costal, yellow=pectoral girdle and forelimb, and purple=pelvic girdle and hindlimb. Scale bar equals 10 cm.


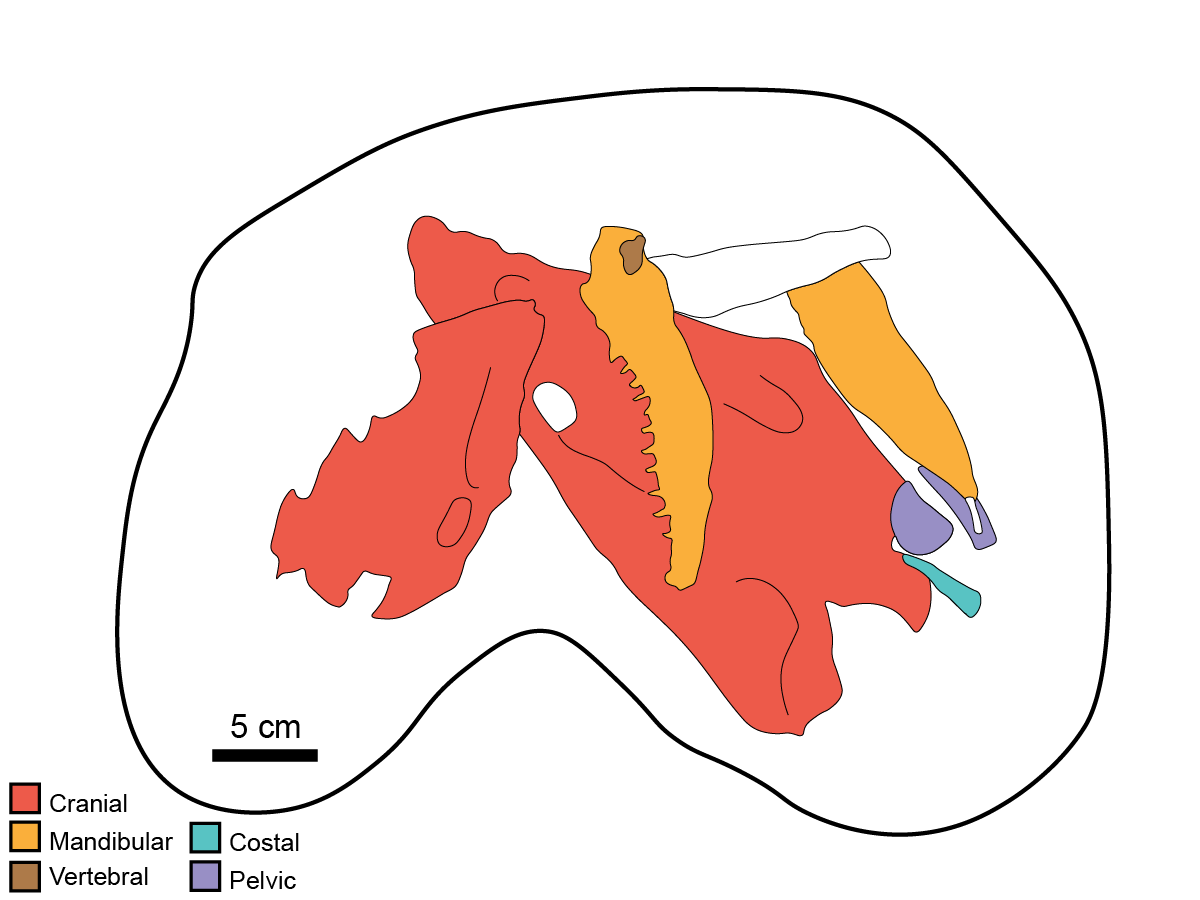

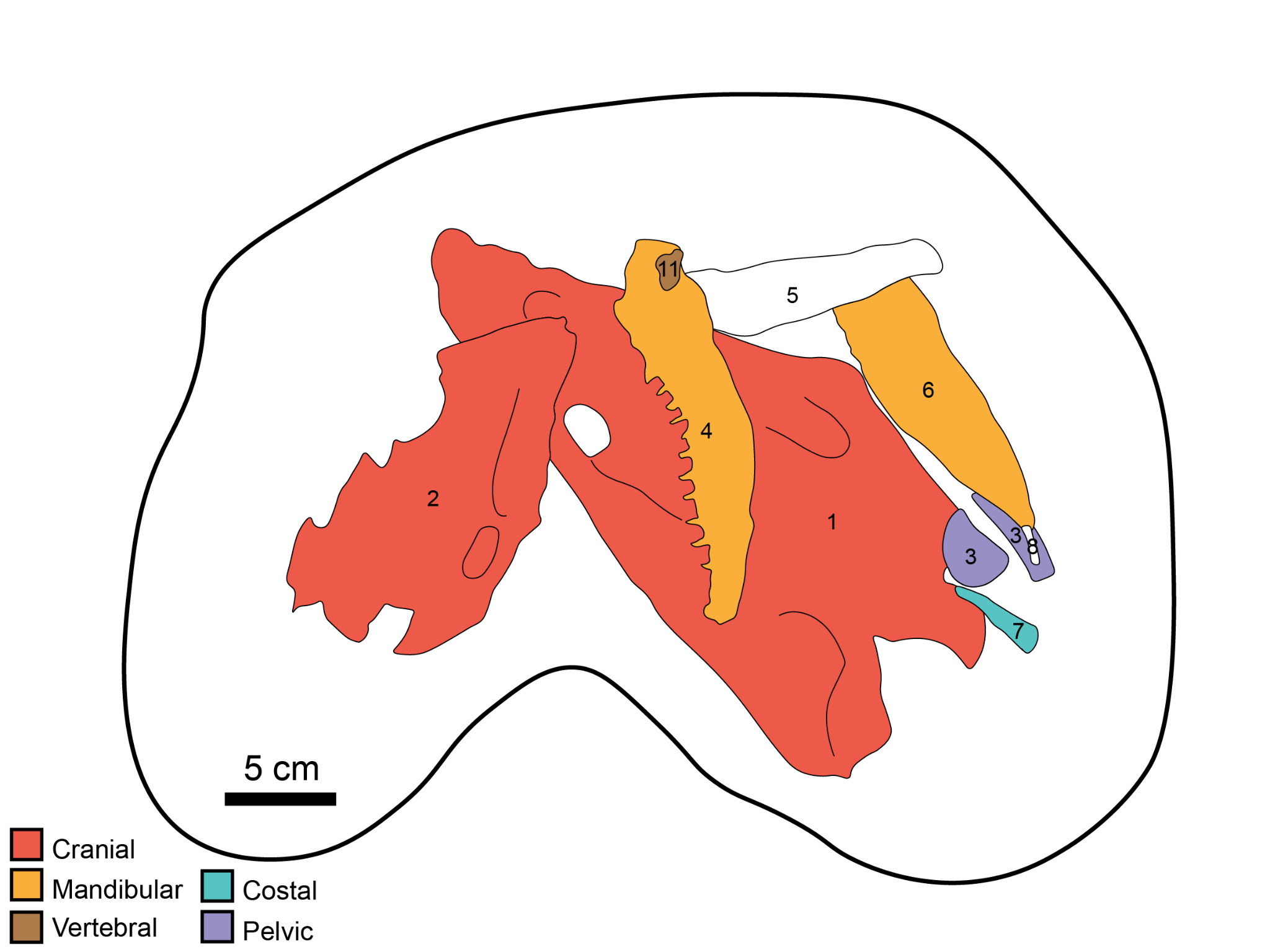


**S2 Fig. Schematic drawing of field jacket NK16 J3 2-3.** High resolution jacket map as included in quarry map (top) and with numbers that correspond to “Prep Lab Number” in S1 Table (bottom). Note that two additional elements of this jacket are present but could not be mapped. Colors indicate anatomical position as follows: red=cranial, orange=mandibular, brown=vertebral, blue=costal, and purple=pelvic girdle and hindlimb. Scale bar equals 5 cm.


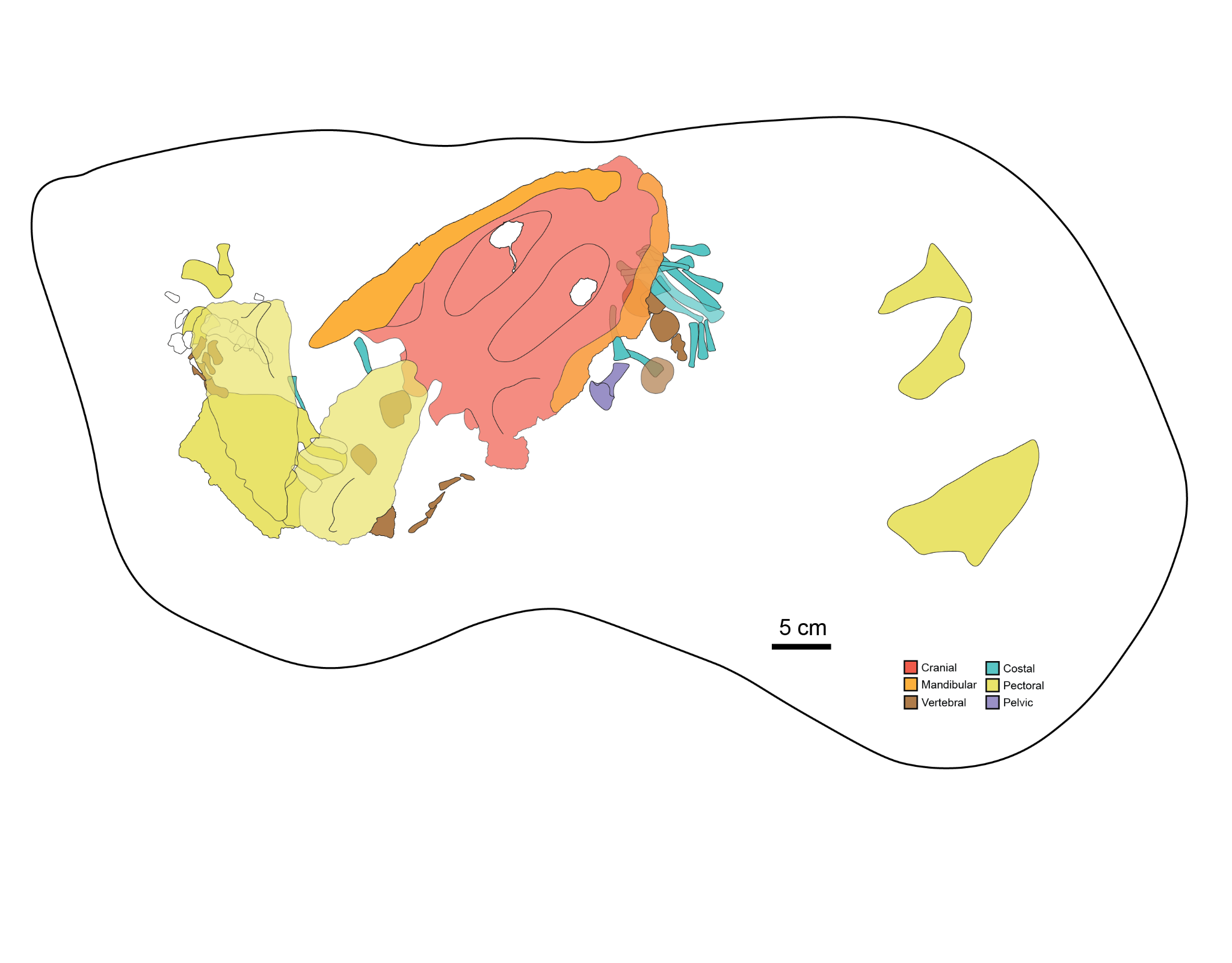

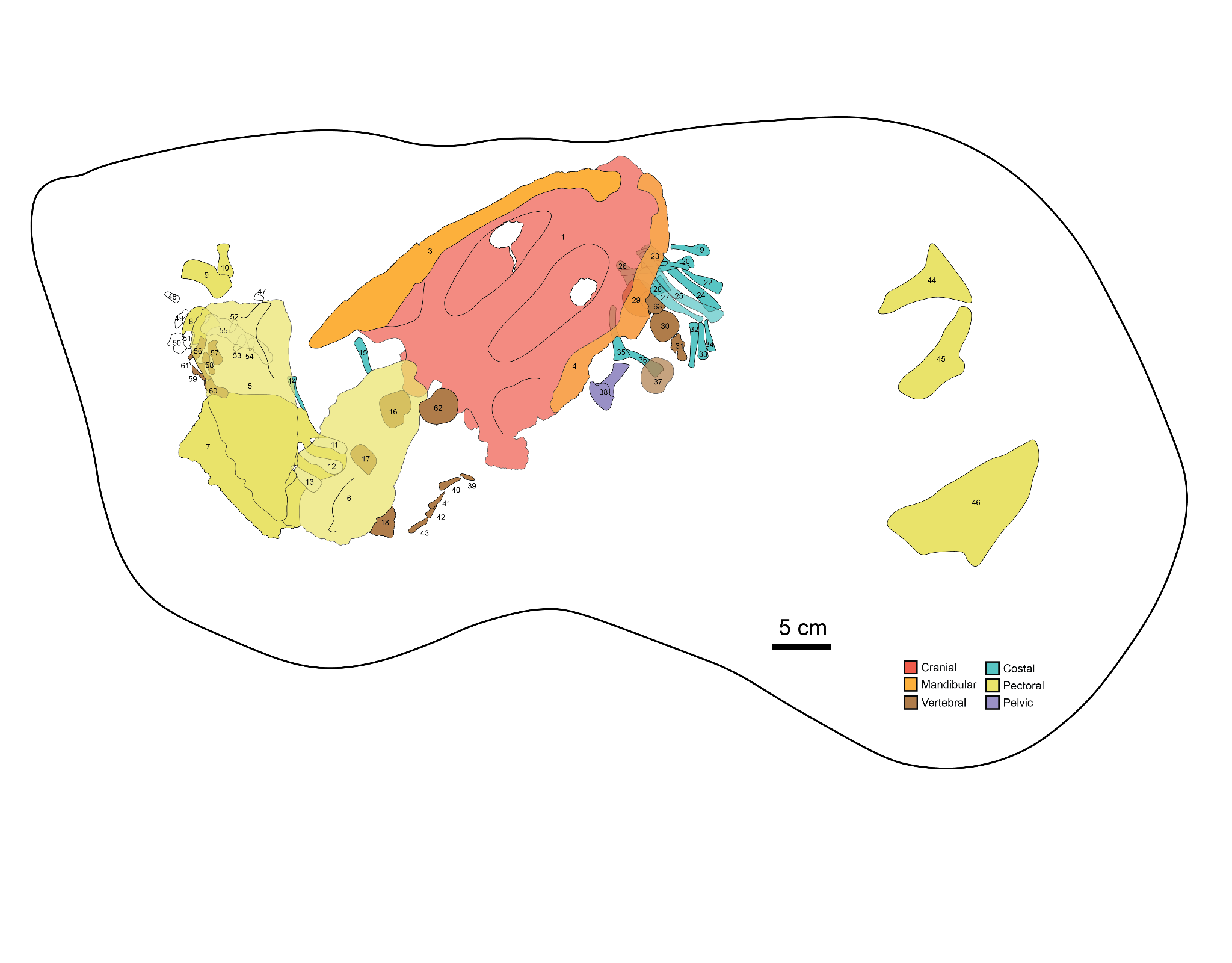


**S3 Fig. Schematic drawing of field jacket NK16 J8 1-2.** High resolution jacket map as included in quarry map (top) and with numbers that correspond to “Prep Lab Number” in S1 Table (bottom). Note that additional elements of this jacket are present but could not be mapped. Some elements are slightly transparent to show underlying bones used in the azimuthal and/or total element count analyses. Colors indicate anatomical position as follows: red=cranial, orange=mandibular, brown=vertebral, blue=costal, yellow=pectoral girdle and forelimb, and purple=pelvic girdle and hindlimb. Scale bar equals 5 cm.


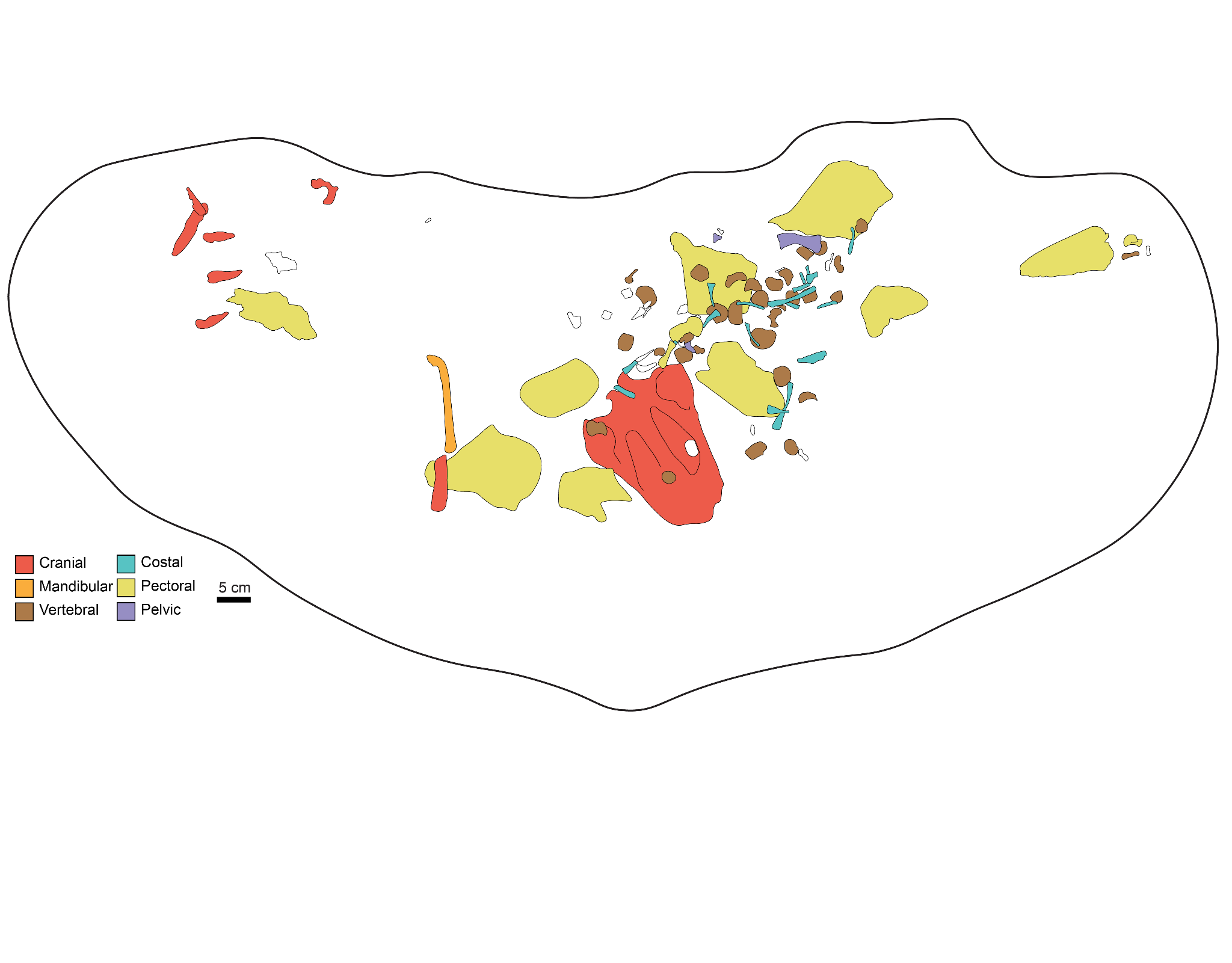

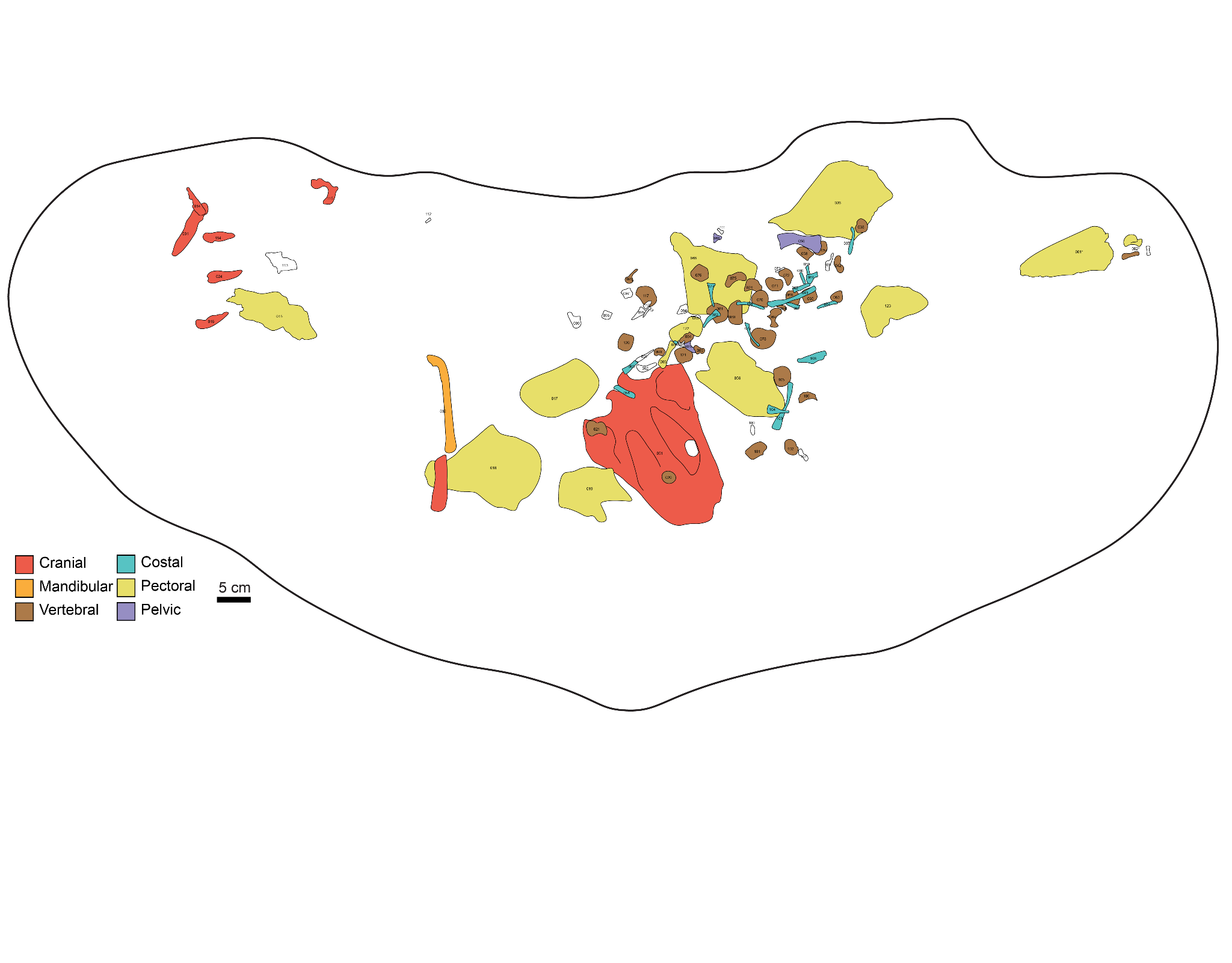


**S4 Fig. Schematic drawing of field jacket NK18-D1-000.** High resolution jacket map as included in quarry map (top) and with numbers that correspond to “Prep Lab Number” in S1 Table (bottom). Colors indicate anatomical position as follows: red=cranial, orange=mandibular, brown=vertebral, blue=costal, yellow=pectoral girdle and forelimb, and purple=pelvic girdle and hindlimb. Scale bar equals 5 cm.


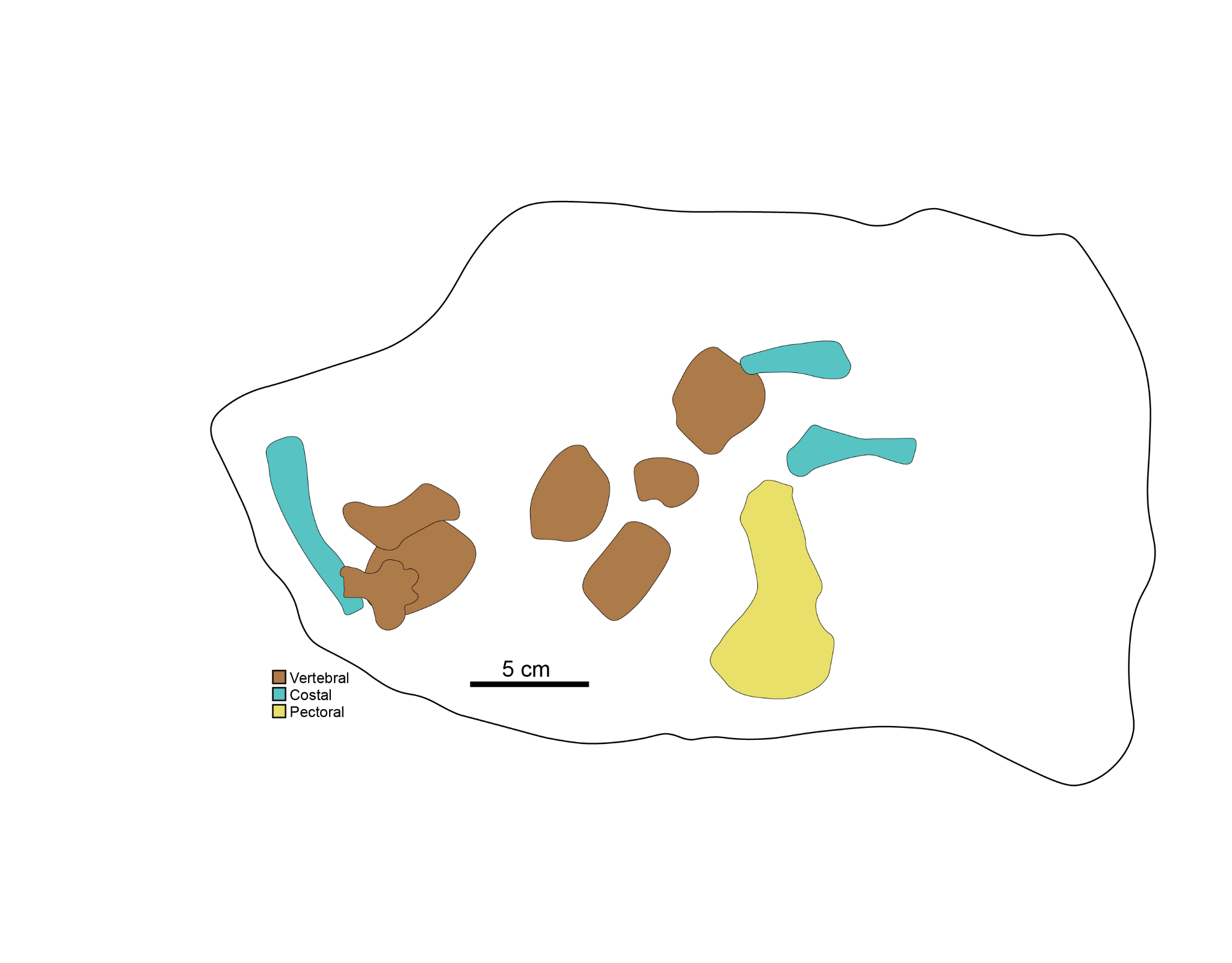

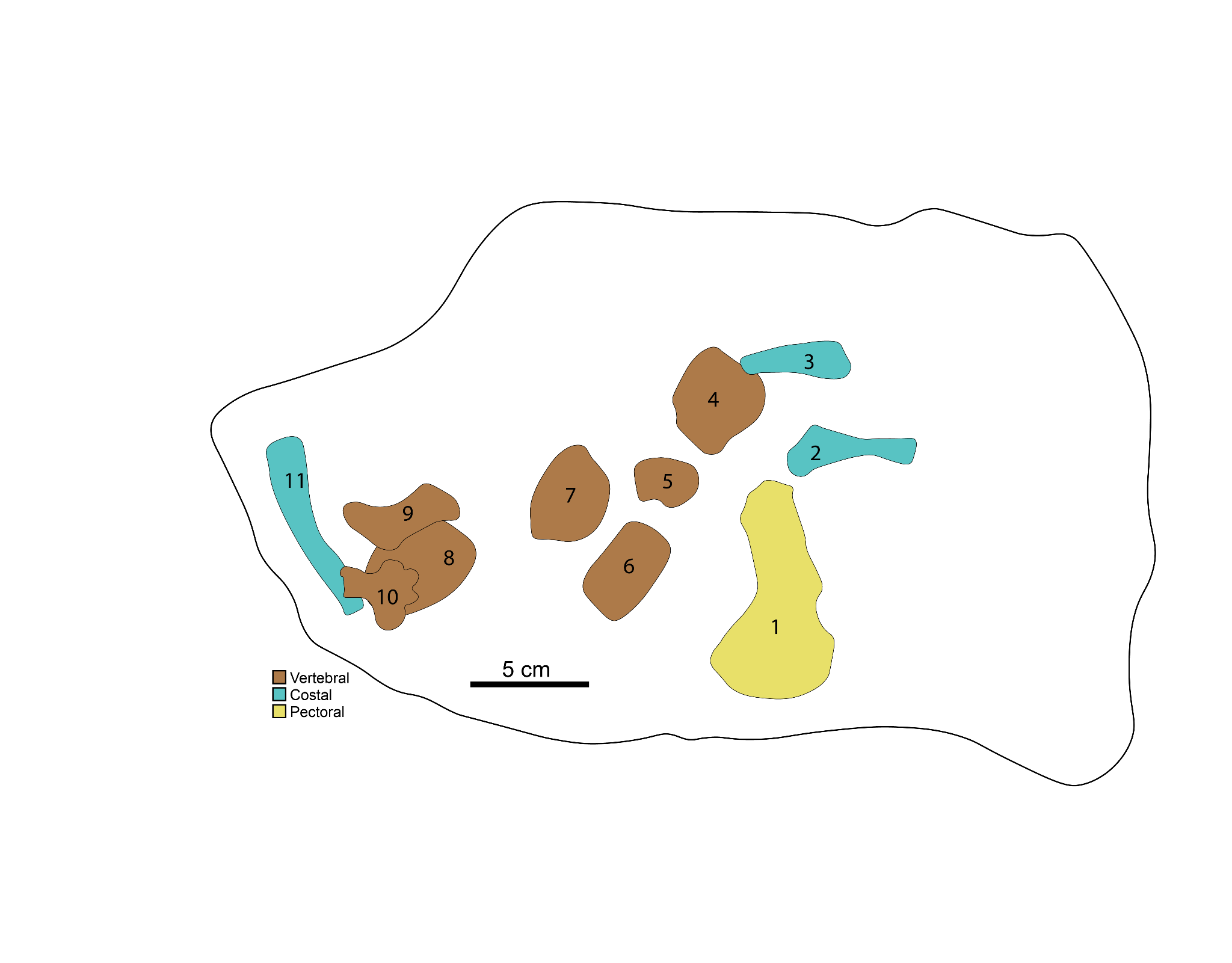


**S5 Fig. Schematic drawing of field jacket NK19-A2-706.21.** High resolution jacket map as included in quarry map (top) and with numbers that correspond to “Prep Lab Number” in S1 Table (bottom). Colors indicate anatomical position as follows: brown=vertebral, blue=costal, and yellow=pectoral girdle and forelimb. Scale bar equals 5 cm.


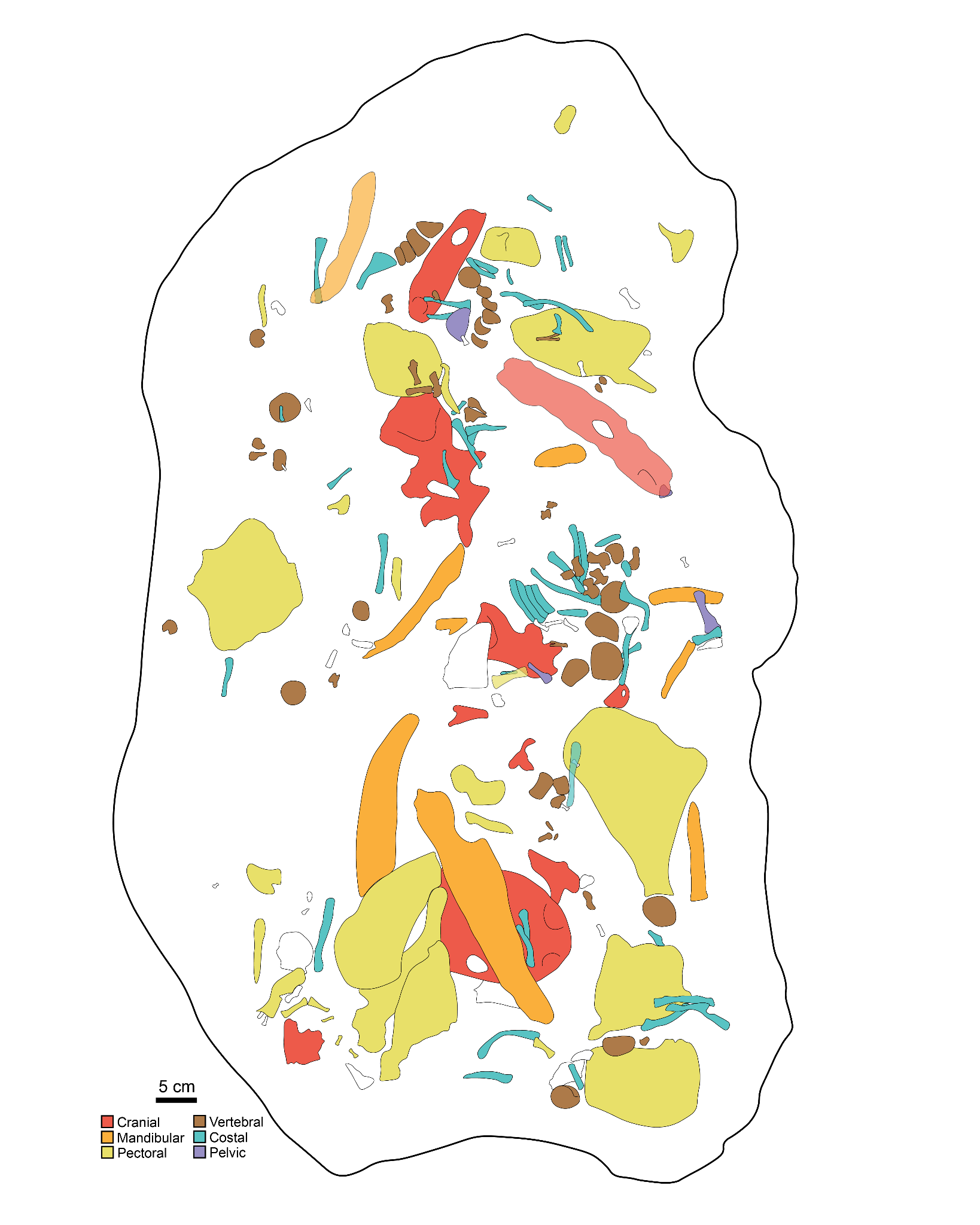

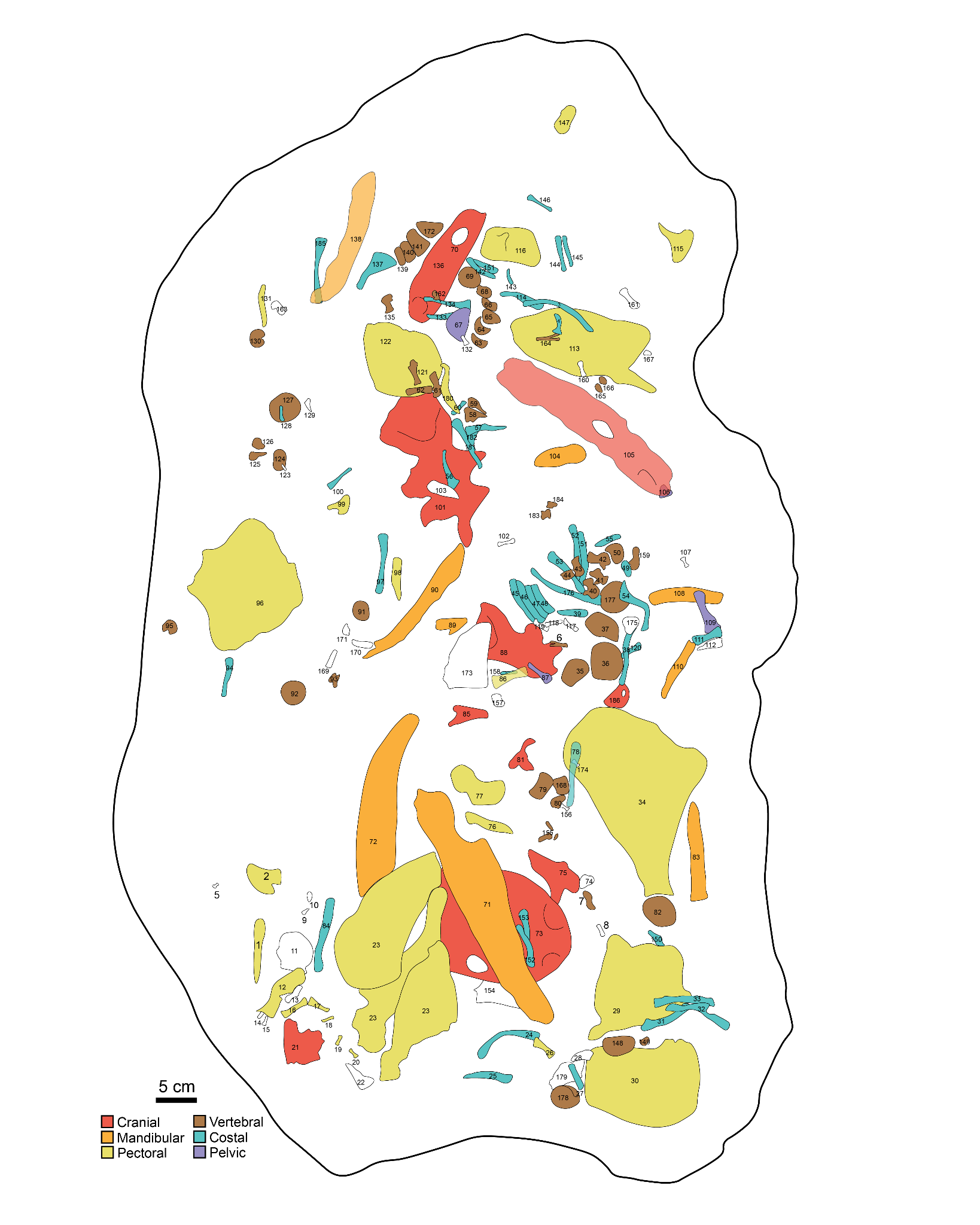


**S6 Fig. Schematic drawing of field jacket NK19-C2-709.3.** High resolution jacket map as included in quarry map (top) and with numbers that correspond to “Prep Lab Number” in S1 Table (bottom). Some elements are slightly transparent to show underlying bones used in the azimuthal and/or total element count analyses. At the time of submission, this jacket was still undergoing preparation, so several skulls are only partially revealed. Colors indicate anatomical position as follows: red=cranial, orange=mandibular, brown=vertebral, blue=costal, yellow=pectoral girdle and forelimb, and purple=pelvic girdle and hindlimb. Scale bar equals 5 cm.


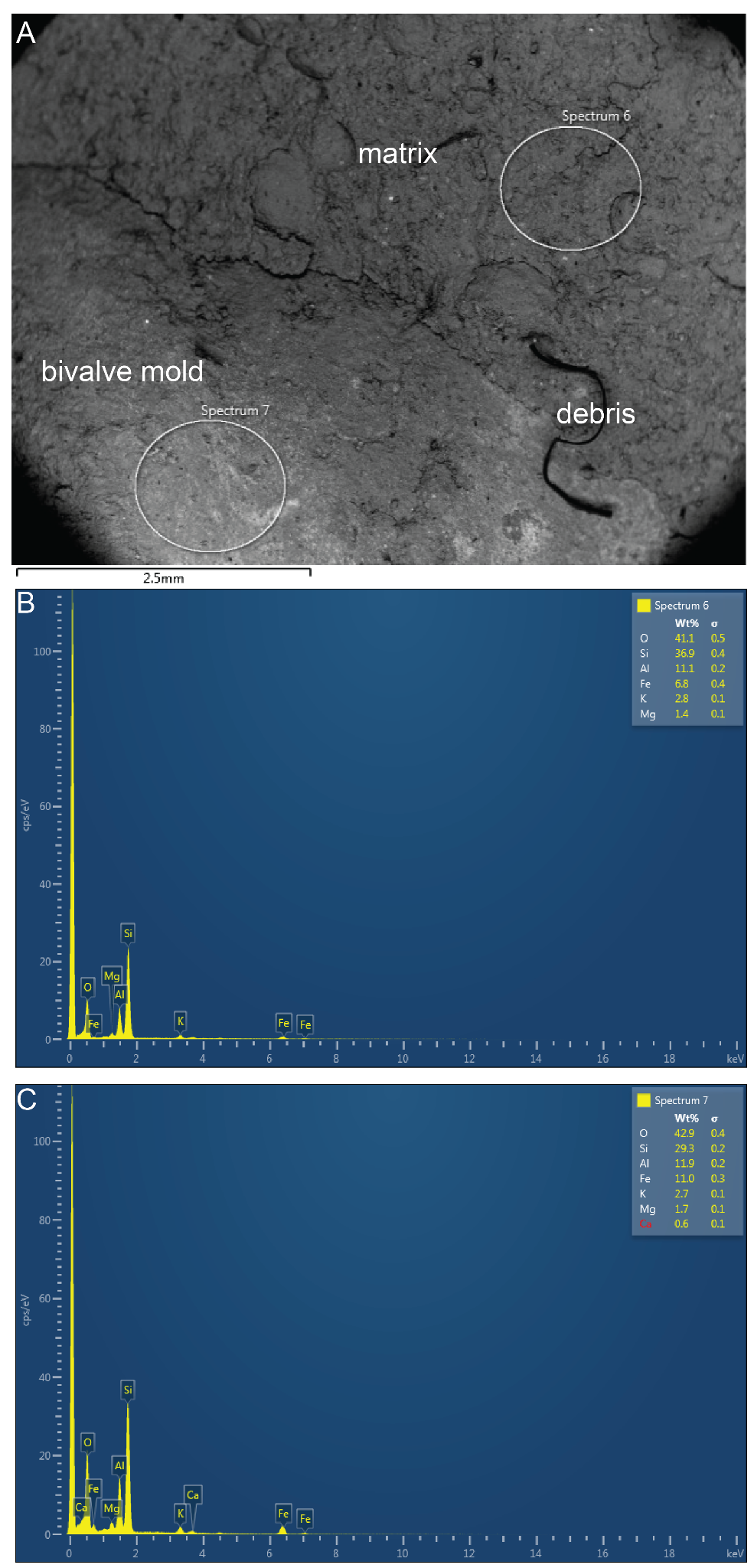


**S7 Fig. Scanning electron microscope (SEM) image and energy dispersive spectroscopy (EDS) X-ray spectra of a bivalve mold and background matrix.** (A) SEM image with circles marking the spots from which spectra were obtained. (B) EDS of the bivalve mold. (C) EDS of the matrix.


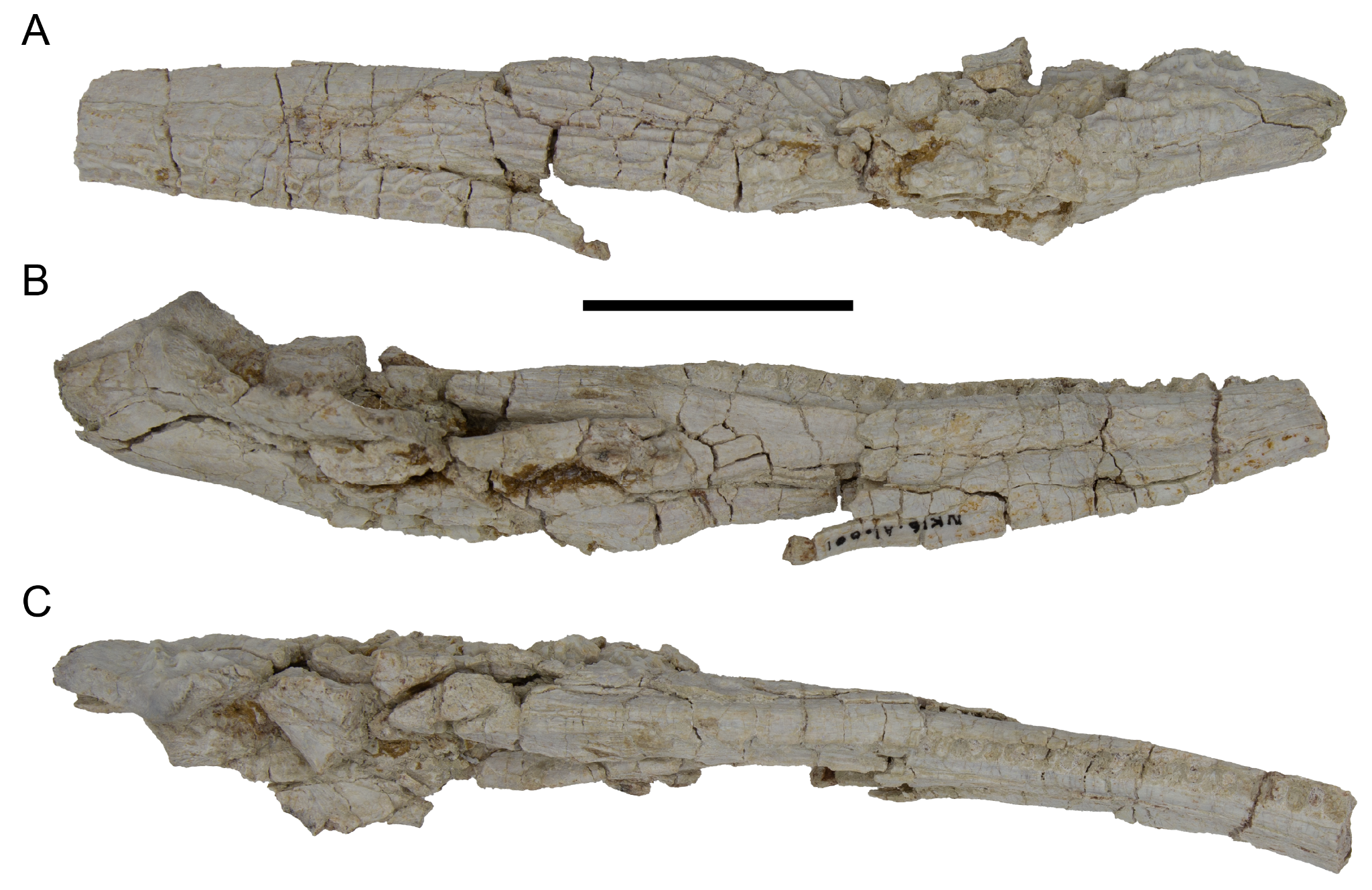


**S8 Fig. Photographs of UWGM 7568, a damaged mandible from the NK bonebed.** (A) labial view, (B) lingual view, and (C) occlusal/dorsal view. Note the crushed glenoid region and the postsplenial split off from the ramus–damage inconsistent with unidirectional and uniform sediment compaction. Scale bar equals 5 cm.

REFERENCES

Benton, M. J. (1985). Classification and phylogeny of the diapsid reptiles. *Zoological Journal of the Linnean Society*, *84*(2), 97–164. https://doi.org/10.1111/j.1096-3642.1985.tb01796.x

Berg, L. S. (1940). *Classification of Fishes, Both Recent and Fossil* (Vol. 5). Akademiia Nauk.

Berry, E. W. (1924). Fossil plants and unios in the red beds of Wyoming. *The Journal of Geology*, *32*(6), 488–497.

Bogan, A. E., & Weaver, P. G. (2012). A new genus and new species of freshwater mussel from the mid Late Triassic rift lakes of eastern North Carolina (Bivalvia: Unionida: Cf. Unionidae). *THE NAUTILUS*, *126*(3), 105–112.

Buffa, V., Jalil, N. E., & Steyer, J. S. (2019). Redescription of *Arganasaurus* (*Metoposaurus*) *azerouali* (Dutuit) comb. Nov. From the Upper Triassic of the Argana Basin (Morocco), and the first phylogenetic analysis of the Metoposauridae (Amphibia, Temnospondyli). *Papers in Palaeontology*, 1–19. https://doi.org/10.1002/spp2.1259

Case, E. C. (1931). Description of a new species of *Buettneria*, with a discussion of the brain case. *Contributions from the Museum of Paleontology University of Michigan*, *3*, 187–206.

Doyle, K. D., & Sues, H.-D. (1995). Phytosaurs (Reptilia: Archosauria) from the Upper Triassic New Oxford Formation of York County, Pennsylvania. *Journal of Vertebrate Paleontology*, *15*(3), 545–553. https://doi.org/10.1080/02724634.1995.10011247

Dutuit, J.-M. (1976). Introduction a l’étude paléontologique du Trias continental Marocain. Description des premiers stegocephales recueillis dans le couloir d’Argana (atlas occidental): Mémoires du Muséum National d’Histoire Naturelle. *Sciences de La Terre*, *36*(C), 1–253.

Gauthier, J., Kluge, A. G., & Rowe, T. (1988). Amniote phylogeny and the importance of fossils. *Cladistics*, *4*(2), 105–209. https://doi.org/10.1111/j.1096-0031.1988.tb00514.x

Gee, B. M., & Kufner, A. M. (2022). Revision of the Late Triassic metoposaurid “*Metoposaurus*” *bakeri* (Amphibia: Temnospondyli) from Texas, USA and a phylogenetic analysis of the Metoposauridae. *PeerJ*, *10*, e14065. https://doi.org/10.7717/peerj.14065

Gibson, S. Z. (2018). A new species of *Lasalichthys* (Actinopterygii, Redfieldiiformes) from the Upper Triassic Dockum Group of Howard County, Texas, with revisions to the genera *Lasalichthys* and *Synorichthys*. *Journal of Vertebrate Paleontology*, *38*(5), e1513009. https://doi.org/10.1080/02724634.2018.1513009

Good, S. C. (1998). Freshwater bivalve fauna of the Late Triassic (Carnian–Norian) Chinle, Dockum, and Dolores formations of the southwest United States. In *Bivalves: An Eon of Evolution* (pp. 223–250). University of Calgary Press.

Goodrich, E. S. (1930). *Studies on the Structure & Development of Vertebrates: Vol. XXX*. Macmillan and Co.

Gray, J. E. (1854). A revision of the arrangement of the families of bivalve shells (Conchifera). *The Annals and Magazine of Natural History; Zoology, Botany, and Geology*, *13*(73–78), 408–418.

Huene, F. von. (1946). Die großen Stämme der Tetrapoden in den geologischen Zeiten. *Biologisches Zentralblatt*, *65*, 268–275.

Hutchinson, P. (1973). A revision of the redfieldiiform and perleidiform fishes from the Triassic of Bekkers Kraal (South Africa) and Brookvale (New South Wales). *Bulletin of the British Museum (Natural History) Geology*, *22*(3), 233–254.

Huxley, T. H. (1880). On the application of the laws of evolution to the arrangement of the Vertebrata, and more particularly of the Mammalia. In *Proceedings of the Zoological Society of London* (pp. 649–662). The Zoological Society of London by Academic Press. https://hdl.handle.net/2027/uc1.c026289462

Jaekel, O. (1909). Über die Klassen der Tetrapoden. *Zoologischer Anzeiger*, *34*, 193–212.

Kammerer, C. F., Butler, R. J., Bandyopadhyay, S., & Stocker, M. R. (2016). Relationships of the Indian phytosaur *Parasuchus hislopi* Lydekker, 1885. *Papers in Palaeontology*, *2*(1), 1–23. https://doi.org/10.1002/spp2.1022

Konietzko-Meier, D., Teschner, E. M., Bodzioch, A., & Sander, P. M. (2020). Pentadactyl manus of the *Metoposaurus krasiejowensis* from the Late Triassic of Poland, the first record of pentadactyly among Temnospondyli. *Journal of Anatomy*, *237*(6), 1151–1161. https://doi.org/10.1111/joa.13276

Lucas, S. G., Rinehart, L. F., Heckert, A. B., Hunt, A. P., & Spielmann, J. A. (2016). Rotten Hill: A Late Triassic bonebed in the Texas Panhandle, USA. *New Mexico Museum of Natural History & Science*, *72*.

Lucas, S. G., Rinehart, L. F., Krainer, K., Spielmann, J. A., & Heckert, A. B. (2010). Taphonomy of the Lamy amphibian quarry: A Late Triassic bonebed in New Mexico, U.S.A. *Palaeogeography, Palaeoclimatology, Palaeoecology*, *298*(3–4), 388–398. https://doi.org/10.1016/j.palaeo.2010.10.025

Lydekker, R. (1885). *The Reptilia & Amphibia of the Maleri & Denwa Groups*. Geological Survey Office.

Marsh, A. D., Parker, W. G., Nesbitt, S. J., Kligman, B. T., & Stocker, M. R. (2022). *Puercosuchus traverorum* n. gen. n. sp.: A new malerisaurine azendohsaurid (Archosauromorpha: Allokotosauria) from two monodominant bonebeds in the Chinle Formation (Upper Triassic, Norian) of Arizona. *Journal of Paleontology*, *96*(S90), 1–39. https://doi.org/10.1017/jpa.2022.49

Marshall, W. B. (1929). New fossil land and fresh-water mollusks from the Reynosa Formation of Texas. *Proceeding of the United States National Museum*, *76*, 1–6.

Meyer, H. von. (1861). Reptilien aus dem Stubensandstein des oberen Keupers. *Palaeontographica*, *7*, 253–346.

Nelson, J. S., Grande, T., & Wilson, M. V. H. (2016). *Fishes of the world* (Fifth edition). John Wiley & Sons.

Nesbitt, S. J. (2011). The early evolution of archosaurs: Relationships and the origin of major clades. *Bulletin of the American Museum of Natural History*, *352*, 1–292. https://doi.org/10.1206/352.1

Nesbitt, S. J., Stocker, M. R., Ezcurra, M. D., Fraser, N. C., Heckert, A. B., Parker, W. G., Mueller, B., Sengupta, S., Bandyopadhyay, S., Pritchard, A. C., & Marsh, A. D. (2022). Widespread azendohsaurids (Archosauromorpha, Allokotosauria) from the Late Triassic of western USA and India. *Papers in Palaeontology*, *8*(1). https://doi.org/10.1002/spp2.1413

Newell, N. D. (1965). Classification of the Bivalvia. *American Museum Novitates*, *2206*.

Osborn, H. F. (1903). On the primary division of the Reptilia into two sub-classes, Synapsida and Diapsida. *Science*, *17*(424), 275–276.

Rinehart, L. F., & Lucas, S. G. (2016). *Eocyclotosaurus appetolatus*, a Middle Triassic amphibian: Osteology, life history, and paleobiology. *New Mexico Museum of Natural History & Science Bulletin*, *70*, 1–117.

Rinehart, L. F., Lucas, S. G., & Heckert, A. B. (2024). Lamy amphibian quarry, a Late Triassic metoposaur-dominated bonebed in New Mexico. *New Mexico Museum of Natural History and Science Bulletin*, *96*.

Schaeffer, B. (1984). On the relationships of the Triassic-Liassic redfieldiiform fishes. *American Museum Novitates*, *2795*, 1–18.

Schoch, R. R. (2013). The evolution of major temnospondyl clades: An inclusive phylogenetic analysis. *Journal of Systematic Palaeontology*, *11*(6), 673–705. https://doi.org/10.1080/14772019.2012.699006

Simpson, C. T. (1895). Description of four new Triassic Unios from the Staked Plains of Texas. *Proceeding of the United States National Museum*, *18*(1072), 381–385.

Sulej, T. (2007). Osteology, variability, and evolution of *Metoposaurus*, a temnospondyl from the Late Triassic of Poland. *Palaeontologia Polonica*, *64*, 29–139.

von Zittel, K. A. (1887). *Handbuch der Paläeontologie. Abteilung 1. Paläozoologie. Band III. Vertebrata (Pisces, Amphibia, Reptilia, Aves)*. Munich and Leipzig.

Watson, D. M. S. (1919). The structure, evolution and origin of the Amphibia. - The orders Rachitomi and Stereospondyli. *Philosophical Transactions of the Royal Society of London. Series B, Biological Sciences*, *209*(360), 1–73. https://doi.org/10.2307/j.ctt211qv60.7

Woodward, A. S. (1891). *Catalogue of Fossil Fishes in the British Museum (Natural History). Part II.* British Museum (Natural History).

Zieritz, A., Sartori, A. F., Bogan, A. E., & Aldridge, D. C. (2015). Reconstructing the evolution of umbonal sculptures in the Unionida. *Journal of Zoological Systematics and Evolutionary Research*, *53*(1), 76–86. https://doi.org/10.1111/jzs.12077
