# Supplementary material for: A new metoposaurid (Temnospondyli) bonebed from the lower Popo Agie Formation (Carnian, Triassic) and an assessment of skeletal sorting
Source: PLoS One. 2025 Apr 2;20(4):e0317325. doi: 10.1371/journal.pone.0317325 (PMC11964259; doi:10.1371/journal.pone.0317325)
Supplement: Attachment — Submitted filename: Response to PONE Reviewer Comments.docx [file pone.0317325.s008.docx]

PONE-D-24-59951

A new metoposaurid (Temnospondyli) bonebed from the lower Popo Agie Formation (Carnian, Triassic) and an assessment of skeletal sorting in temnospondyls

PLOS ONE

When submitting your revision, we need you to address these additional requirements.

1.Please ensure that your manuscript meets PLOS ONE's style requirements, including those for file naming. The PLOS ONE style templates can be found at

[https://journals.plos.org/plosone/s/file?id=wjVg/PLOSOne_formatting_sample_main_body.pdf](https://urldefense.com/v3/__https:/journals.plos.org/plosone/s/file?id=wjVg*PLOSOne_formatting_sample_main_body.pdf__;Lw!!Mak6IKo!NdKyPMbkUUspxF3VVkhGC2r8vUOG8GDEUjm-GP6051HlXCs4GHeJ8YSPXm53rQBadMwHXdy1mDgTtcWT$) and

[https://journals.plos.org/plosone/s/file?id=ba62/PLOSOne_formatting_sample_title_authors_affiliations.pdf](https://urldefense.com/v3/__https:/track.editorialmanager.com/CL0/https:*2F*2Fjournals.plos.org*2Fplosone*2Fs*2Ffile*3Fid=ba62*2FPLOSOne_formatting_sample_title_authors_affiliations.pdf/1/010f01948316cd17-0bb8f520-beb0-4937-b766-bf1e71c504c1-000000/uU4lHV5KXB41lpxjDihgfWv6KfCtzhJr1isyhj1KHy4=194__;JSUlJSUlJQ!!Mak6IKo!NdKyPMbkUUspxF3VVkhGC2r8vUOG8GDEUjm-GP6051HlXCs4GHeJ8YSPXm53rQBadMwHXdy1mKXmm5KS$)

Our manuscript meets the style requirements of PLOS ONE including file names.

2. Thank you for stating the following financial disclosure:

“A David B. Jones Foundation grant awarded to DML helped fund the excavation of this material.”

Please state what role the funders took in the study.  If the funders had no role, please state: "The funders had no role in study design, data collection and analysis, decision to publish, or preparation of the manuscript."

If this statement is not correct you must amend it as needed.

Please include this amended Role of Funder statement in your cover letter; we will change the online submission form on your behalf.

The financial disclosure should read as follows: “A David B. Jones Foundation grant awarded to DML helped fund the excavation of this material. The funders had no role in study design, data collection and analysis, decision to publish, or preparation of the manuscript.”

3. Your ethics statement should only appear in the Methods section of your manuscript. If your ethics statement is written in any section besides the Methods, please move it to the Methods section and delete it from any other section. Please ensure that your ethics statement is included in your manuscript, as the ethics statement entered into the online submission form will not be published alongside your manuscript.

The ethics statement present in the online submissions form was added to the Methods section of the manuscript.

4. We note that Figures 1-3 in your submission contain map/satellite images which may be copyrighted. All PLOS content is published under the Creative Commons Attribution License (CC BY 4.0), which means that the manuscript, images, and Supporting Information files will be freely available online, and any third party is permitted to access, download, copy, distribute, and use these materials in any way, even commercially, with proper attribution. For these reasons, we cannot publish previously copyrighted maps or satellite images created using proprietary data, such as Google software (Google Maps, Street View, and Earth). For more information, see our copyright guidelines: [http://journals.plos.org/plosone/s/licenses-and-copyright](https://urldefense.com/v3/__http:/journals.plos.org/plosone/s/licenses-and-copyright__;!!Mak6IKo!NdKyPMbkUUspxF3VVkhGC2r8vUOG8GDEUjm-GP6051HlXCs4GHeJ8YSPXm53rQBadMwHXdy1mOv33GNl$).

 We require you to either (a) present written permission from the copyright holder to publish these figures specifically under the CC BY 4.0 license, or (b) remove the figures from your submission:

a. You may seek permission from the original copyright holder of Figures 1-3 to publish the content specifically under the CC BY 4.0 license.

 We recommend that you contact the original copyright holder with the Content Permission Form ([http://journals.plos.org/plosone/s/file?id=7c09/content-permission-form.pdf](https://urldefense.com/v3/__http:/journals.plos.org/plosone/s/file?id=7c09*content-permission-form.pdf__;Lw!!Mak6IKo!NdKyPMbkUUspxF3VVkhGC2r8vUOG8GDEUjm-GP6051HlXCs4GHeJ8YSPXm53rQBadMwHXdy1mEtv3Xio$)) and the following text:

“I request permission for the open-access journal PLOS ONE to publish XXX under the Creative Commons Attribution License (CCAL) CC BY 4.0 ([http://creativecommons.org/licenses/by/4.0/](https://urldefense.com/v3/__http:/creativecommons.org/licenses/by/4.0/__;!!Mak6IKo!NdKyPMbkUUspxF3VVkhGC2r8vUOG8GDEUjm-GP6051HlXCs4GHeJ8YSPXm53rQBadMwHXdy1mBvIYBnG$)). Please be aware that this license allows unrestricted use and distribution, even commercially, by third parties. Please reply and provide explicit written permission to publish XXX under a CC BY license and complete the attached form.”

 Please upload the completed Content Permission Form or other proof of granted permissions as an "Other" file with your submission.

 In the figure caption of the copyrighted figure, please include the following text: “Reprinted from [ref] under a CC BY license, with permission from [name of publisher], original copyright [original copyright year].”

Figure 1 Map data and silhouettes: we added this to the figure caption “Redfieldiid silhouette by AMK and archosauromorph silhouette by DML. *Chinlestegophis* (stand in for *Ninumbeehan*) silhouette in the public domain by T.M. Keesey. Other silhouettes used under Creative Commons Attribution 3.0 Unported <https://creativecommons.org/licenses/by/3.0/> from phylopic.org. Metoposaurid silhouette after D. Bogdanov and phytosaur silhouette by S.A. Hartman. Geological map and outcrop extent of Chugwater Group data (B) from https://macrostrat.org API under a CC-BY-4.0 license. The North America silhouette and state outlines (B) based on 'USA_States_Generalized' and 'Territorial_Evolution_1867_-2003_TE_' shapefiles in the public domain (Sources: Esri; U.S. Department of Commerce, Census Bureau; U.S. Department of Commerce (DOC), National Oceanic and Atmospheric Administration (NOAA), National Ocean Service (NOS), National Geodetic Survey (NGS). The paleogeographic map outline of Pangea was modified after [(64)](https://www.zotero.org/google-docs/?wcXStb). Paleoclimate map overlay modified after [(65)](https://www.zotero.org/google-docs/?UyLoYY).”

Figures 2 and 3 are entirely our own work.

 b. If you are unable to obtain permission from the original copyright holder to publish these figures under the CC BY 4.0 license or if the copyright holder’s requirements are incompatible with the CC BY 4.0 license, please either i) remove the figure or ii) supply a replacement figure that complies with the CC BY 4.0 license. Please check copyright information on all replacement figures and update the figure caption with source information. If applicable, please specify in the figure caption text when a figure is similar but not identical to the original image and is therefore for illustrative purposes only.

The following resources for replacing copyrighted map figures may be helpful:

 USGS National Map Viewer (public domain): [http://viewer.nationalmap.gov/viewer/](https://urldefense.com/v3/__http:/viewer.nationalmap.gov/viewer/__;!!Mak6IKo!NdKyPMbkUUspxF3VVkhGC2r8vUOG8GDEUjm-GP6051HlXCs4GHeJ8YSPXm53rQBadMwHXdy1mMGSI6E9$)

The Gateway to Astronaut Photography of Earth (public domain): [http://eol.jsc.nasa.gov/sseop/clickmap/](https://urldefense.com/v3/__http:/eol.jsc.nasa.gov/sseop/clickmap/__;!!Mak6IKo!NdKyPMbkUUspxF3VVkhGC2r8vUOG8GDEUjm-GP6051HlXCs4GHeJ8YSPXm53rQBadMwHXdy1mJOAaWY5$)

Maps at the CIA (public domain): [https://www.cia.gov/library/publications/the-world-factbook/index.html](https://urldefense.com/v3/__https:/www.cia.gov/library/publications/the-world-factbook/index.html__;!!Mak6IKo!NdKyPMbkUUspxF3VVkhGC2r8vUOG8GDEUjm-GP6051HlXCs4GHeJ8YSPXm53rQBadMwHXdy1mCyJDeWt$) and [https://www.cia.gov/library/publications/cia-maps-publications/index.html](https://urldefense.com/v3/__https:/www.cia.gov/library/publications/cia-maps-publications/index.html__;!!Mak6IKo!NdKyPMbkUUspxF3VVkhGC2r8vUOG8GDEUjm-GP6051HlXCs4GHeJ8YSPXm53rQBadMwHXdy1mBIPsuEQ$)

NASA Earth Observatory (public domain): [http://earthobservatory.nasa.gov/](https://urldefense.com/v3/__http:/earthobservatory.nasa.gov/__;!!Mak6IKo!NdKyPMbkUUspxF3VVkhGC2r8vUOG8GDEUjm-GP6051HlXCs4GHeJ8YSPXm53rQBadMwHXdy1mBRN9dH5$)

Landsat: [http://landsat.visibleearth.nasa.gov/](https://urldefense.com/v3/__http:/landsat.visibleearth.nasa.gov/__;!!Mak6IKo!NdKyPMbkUUspxF3VVkhGC2r8vUOG8GDEUjm-GP6051HlXCs4GHeJ8YSPXm53rQBadMwHXdy1mG3fPtEC$)

USGS EROS (Earth Resources Observatory and Science (EROS) Center) (public domain): [http://eros.usgs.gov/#](https://urldefense.com/v3/__http:/eros.usgs.gov/*__;Iw!!Mak6IKo!NdKyPMbkUUspxF3VVkhGC2r8vUOG8GDEUjm-GP6051HlXCs4GHeJ8YSPXm53rQBadMwHXdy1mHL8AmDF$)

Natural Earth (public domain): [http://www.naturalearthdata.com/](https://urldefense.com/v3/__http:/track.editorialmanager.com/CL0/http:*2F*2Fwww.naturalearthdata.com*2F/1/010f01948316cd17-0bb8f520-beb0-4937-b766-bf1e71c504c1-000000/Vm4KlKsz9unpLg5RptfAsQcFyjjvGydIbA0Y8UMYUjo=194__;JSUl!!Mak6IKo!NdKyPMbkUUspxF3VVkhGC2r8vUOG8GDEUjm-GP6051HlXCs4GHeJ8YSPXm53rQBadMwHXdy1mF0BEATk$)

5. We are unable to open your Supporting Information file “Copy of S2 File.R”. Please kindly revise as necessary and re-upload.

This file can be opened in any text editing software and can be run in R.

[Note: HTML markup is below. Please do not edit.]

Reviewers' comments:

Reviewer's Responses to Questions

**Comments to the Author**

1. Is the manuscript technically sound, and do the data support the conclusions?

The manuscript must describe a technically sound piece of scientific research with data that supports the conclusions. Experiments must have been conducted rigorously, with appropriate controls, replication, and sample sizes. The conclusions must be drawn appropriately based on the data presented.

Reviewer #1: Yes

Reviewer #2: Partly

2. Has the statistical analysis been performed appropriately and rigorously?

Reviewer #1: Yes

Reviewer #2: N/A

3. Have the authors made all data underlying the findings in their manuscript fully available?

The [PLOS Data policy](https://urldefense.com/v3/__http:/track.editorialmanager.com/CL0/http:*2F*2Fwww.plosone.org*2Fstatic*2Fpolicies.action*23sharing/1/010f01948316cd17-0bb8f520-beb0-4937-b766-bf1e71c504c1-000000/fvBtPq7QN7wg_FXBqcW540P8g2OpfbIELWHNkVU8NzE=194__;JSUlJSU!!Mak6IKo!NdKyPMbkUUspxF3VVkhGC2r8vUOG8GDEUjm-GP6051HlXCs4GHeJ8YSPXm53rQBadMwHXdy1mKPZCQft$) requires authors to make all data underlying the findings described in their manuscript fully available without restriction, with rare exception (please refer to the Data Availability Statement in the manuscript PDF file). The data should be provided as part of the manuscript or its supporting information, or deposited to a public repository. For example, in addition to summary statistics, the data points behind means, medians and variance measures should be available. If there are restrictions on publicly sharing data—e.g. participant privacy or use of data from a third party—those must be specified.

Reviewer #1: Yes

Reviewer #2: Yes

4. Is the manuscript presented in an intelligible fashion and written in standard English?

PLOS ONE does not copyedit accepted manuscripts, so the language in submitted articles must be clear, correct, and unambiguous. Any typographical or grammatical errors should be corrected at revision, so please note any specific errors here.

Reviewer #1: Yes

Reviewer #2: Yes

5. Review Comments to the Author

Please use the space provided to explain your answers to the questions above. You may also include additional comments for the author, including concerns about dual publication, research ethics, or publication ethics. (Please upload your review as an attachment if it exceeds 20,000 characters)

Reviewer #1: This manuscript is a valuable contribution to bonebed taphonomy in that it addresses the long-time problem of uncontrolled and poorly documented fieldwork done my numerous short-stay field teams in the Late Triassic strata of Wyoming most of which have not been subjected to modern detailed taphonomic analysis. For the first time the authors have recorded detailed sedimentological and taphonomc data at the site as well as collecting large in-situ blocks of the bonebed for controlled mechanical preparation to further their in-depth documentation in the laboratory.
The manuscript deals with the palaeoenvironmental setting first then the bonebed genesis - giving equal emphasis to both fields. Then coming together in the discussion to formulate an evidence-supported taphonomic pathway.
The grammar is good- I have made several suggestions for improvements for clarity.

We have incorporated the majority of these suggestions for clarity/grammar.

One aspect that I found a bit unnecessary for what is essentially a taphonomic rather than a palaeobiological or taxonomic paper was the systematics section. I suggest you retain the species lists but move the descriptions of taxonomically significant anatomical features to supplementary.

We agree and moved the systematic paleontology section from the main text to S1 File. We also added short descriptions of types of fossils present at NK to the “Fossil occurrences” section just before the Discussion.

Lastly I would have like to see a taphonomic pathway diagram in your discussion of your most likely scenario of biogenically-induced mass mortality. It is all there, but it just needs a bit more confidence in the interpretation of your findings.

We think this is an excellent idea and have made a figure (Fig 10) to show a summary of our interpretation of the taphonomic pathway for the NK site.

All-in-all a very good paper for the ever growing community of bonebed specialists, and a very researchworthy paper for Plos One.

Reviewer #2: This is an important article that documents a significant Late Triassic amphibian bonebed in Wyoming and evaluates its taphonomy. It needs some revision to improve the presentation, particularly the comparisons to other Triassic amphibian bonebeds. I also don’t understand the claim that somehow the Voorhies group assignments of the metopo bones are being improved on here by skeletal census? The bones are assigned to Voorhies groups by their perceived hydrodynamic qualities, not by their relative abundance. My specific comments are keyed to Comment indicators on the ms pdf:

Spencer G. Lucas

1. Lines 82-83 What does “subjective” mean here?—all of these analyses are subjective unless bones are actually being put into flumes, yes? So, how is the approach used here any less subjective than earlier work?

As stated in the sentence, the reassignment of skeletal elements to “Voorhies groups” that lack experimental data is inherently subjective. Our skeletal completeness assessment (Fig. 4A–C) simply does not assume the relative dispersal potential of any element in a temnospondyl skeleton.

2. Line 197 The very recent monograph by Rinehart et al. (2024) NMMNH Bulletin 96 should be cited as well. It is up on my ResearchGate page, free download.

The study suggested here was already cited both in the text (line 193 - reference #3) as well as in the table caption (as #3) and in the table (as Rinehart et al., 2024). However, we did note redundancy in a sentence in the caption and have fixed that.

3. Line 334 Given that the bonebed is in pedogenically modified mudrock, why is “channel-lag in a fluvial system” even relevant here? The Lamy bonebed is also in pedogenically modified mudrock and the non-amphibian fossils are rather similar to those in the Wyoming bonebed. So, why is there no more detailed comparison Wyoming-Lamy?

We agree and have specified that the alignments of long bones support no evidence for unidirectional flow in a fluvial system. Additional comparison with Lamy was made in the Discussion and also noted in Reviewer 2’s comment 7.

4. Line 549-609 You mean “Camp Springs” not Santa Rosa, right?

We have clarified proposed equivalence and correlation of the Camp Springs Conglomerate with the Santa Rosa Sandstone/Tecolotito Member of the Santa Rosa Formation in the section “Specimen accessibility, preparation, and photography” on first mention of the Camp Springs Conglomerate.

5. Comparison should be made here to the Eocyclotosaurus bonebed described by Rinehart and Lucas, who also posited it as a debris flow deposit.

This is an excellent point that we had not considered. We have noted this similarity between the EP bonebed and the Eocyclotosaurus bonebed.

6. Lines 675-676 Really? How are the differences explained? Transport must be at play, as well as degree of disarticulation. This needs discussion

We have clarified the phrasing of this sentence to emphasize that the variability of the relative abundance of intermediate dispersal elements between the three sites renders any interpretation of sorting or transport essentially meaningless. Assessing the value of the intermediate dispersal elements with respect to sorting and transport interpretation would require extensive modeling or more sampling of assemblages, both of which are beyond the scope of this study.

7. Line 730 etc. The main weakness of this paper is it does not compare the Wyoming bonebed to the most similar amphibian bonebeds (Lamy, Rotten Hill, Eocyclotosaurus) and instead focuses on the Elkins bonebed, which has never really been analyzed taphonomically and is very different (primarily skulls, in sandstone). I suggest shortening comparison to Elkins and present some meaningful comparisons to the others, particularly Lamy, which is most similar to the Wyoming bonebed.

The comparison to Elkins Place is necessary and an attempt to analyze the site taphonomically given what little data is available compared to more recently excavated metoposaurid sites. Additional comparisons to Lamy and Rotten Hill were added in the “Comparison with other metoposaurid sites” in the Discussion. As noted by the reviewer, the Eocyclotosaurus bonebed is probably in a debris flow and thus not comparable with NK, but it is comparable with EP and noted in the “Elkins Place conundrum” section.

6. PLOS authors have the option to publish the peer review history of their article ([what does this mean?](https://urldefense.com/v3/__https:/track.editorialmanager.com/CL0/https:*2F*2Fjournals.plos.org*2Fplosone*2Fs*2Feditorial-and-peer-review-process*23loc-peer-review-history/1/010f01948316cd17-0bb8f520-beb0-4937-b766-bf1e71c504c1-000000/16JZrNfm0X4gXSpqQMlG8TMa9FUCam4hTosULkM2MK4=194__;JSUlJSUl!!Mak6IKo!NdKyPMbkUUspxF3VVkhGC2r8vUOG8GDEUjm-GP6051HlXCs4GHeJ8YSPXm53rQBadMwHXdy1mHi4O3vG$)). If published, this will include your full peer review and any attached files.

If you choose “no”, your identity will remain anonymous but your review may still be made public.

**Do you want your identity to be public for this peer review?** For information about this choice, including consent withdrawal, please see our [Privacy Policy](https://urldefense.com/v3/__https:/track.editorialmanager.com/CL0/https:*2F*2Fwww.plos.org*2Fprivacy-policy/1/010f01948316cd17-0bb8f520-beb0-4937-b766-bf1e71c504c1-000000/vu7IUCJURwDeUIi-Fc2xGG2SsZWOxsG6zu1WOwZWPZI=194__;JSUl!!Mak6IKo!NdKyPMbkUUspxF3VVkhGC2r8vUOG8GDEUjm-GP6051HlXCs4GHeJ8YSPXm53rQBadMwHXdy1mIUgcPO1$).

Reviewer #1: **Yes:** Roger MH Smith

Reviewer #2: **Yes:** Spencer G. Lucas
